# Melatonin promoted chemotaxins expression in lung epithelial cell stimulated with TNF-α

**DOI:** 10.1186/1465-9921-5-20

**Published:** 2004-11-10

**Authors:** FengMing Luo, XiaoJing Liu, ShuangQing Li, ChunTao Liu, ZengLi Wang

**Affiliations:** 1 West China Hospital of Sichuan University, Chengdu, China

**Keywords:** melatonin, TNF-α, chemotaxin, lung epithelia cell

## Abstract

**Background:**

Patients with asthma demonstrate circadian variations in the airway inflammation and lung function. Pinealectomy reduces the total inflammatory cell number in the asthmatic rat lung. We hypothesize that melatonin, a circadian rhythm regulator, may modulate the circadian inflammatory variations in asthma by stimulating the chemotaxins expression in the lung epithelial cell.

**Methods:**

Lung epithelial cells (A549) were stimulated with melatonin in the presence or absence of TNF-α(100 ng/ml). RANTES (Regulated on Activation Normal T-cells Expressed and Secreted) and eotaxin expression were measured using ELISA and real-time RT-PCR, eosinophil chemotactic activity (ECA) released by A549 was measured by eosinophil chemotaxis assay.

**Results:**

TNF-α increased the expression of RANTES (307.84 ± 33.56 *versus *207.64 ± 31.27 pg/ml of control, p = 0.025) and eotaxin (108.97 ± 10.87 *versus *54.00 ± 5.29 pg/ml of control, p = 0.041). Melatonin(10^-10 ^to 10^-6^M) alone didn't change the expression of RNATES (204.97 ± 32.56 pg/ml) and eotaxin (55.28 ± 6.71 pg/ml). However, In the presence of TNF-α (100 ng/ml), melatonin promoted RANTES (410.88 ± 52.03, 483.60 ± 55.37, 559.92 ± 75.70, 688.42 ± 95.32, 766.39 ± 101.53 pg/ml, treated with 10^-10^, 10^-9^, 10^-8^, 10^-7^,10^-6^M melatonin, respectively) and eotaxin (151.95 ± 13.88, 238.79 ± 16.81, 361.62 ± 36.91, 393.66 ± 44.89, 494.34 ± 100.95 pg/ml, treated with 10^-10^, 10^-9^, 10^-8^, 10^-7^, 10^-6^M melatonin, respectively) expression in a dose dependent manner in A549 cells (compared with TNF-α alone, P < 0.05). The increased release of RANTES and eotaxin in A549 cells by above treatment were further confirmed by both real-time RT-PCR and the ECA assay.

**Conclusion:**

Taken together, our results suggested that melatonin might synergize with pro-inflammatory cytokines to modulate the asthma airway inflammation through promoting the expression of chemotaxins in lung epithelial cell.

## Backgound

Eosinophils are known to be the important effector cells in asthmatic airway inflammations[1]. Previous studies have demonstrated that eosinophils are accumulated in the peripheral blood, the bronchoalveolar lavage fluid, and the airway of the asthmatic patients or the allergen-sensitized animals[2]. Eosinophil trafficking is regulated by a wide variety of chemotactic factors[3]. Eotaxin and RANTES (Regulated on Activation Normal T-cells Expressed and Secreted) are C-C chemotaxins that can recruit eosinophils to the airway in asthma[4]. A variety of tissues and cell types, including lung epithelial cell, produce eotaxin and RANTES which play an important role in airway[5].

Pro-inflammatory cytokines such as tumor necrosis factor (TNF) and interleukin (IL)-1 are released in the early stage of allergic inflammation. In endothelial and epithelial cells, TNF-α induces an influx of eosinophils into tissues through the increased expression of adhesion molecules[6,7]. Although eotaxin and RANTES tend to be expressed constitutively in several cell types, their expression may also be regulated in response to TNF-α in other cell lines[8].

Melatonin(N-acetyl-5-methoxytryptamine) is a key regulator of circadian rhythm homeostasis in humans[9,10]. It also appears to have an important immunomodulatory effect in allergic diseases[11,12]. Melatonin promotes the cytokine production in the peripheral blood mononuclear cell. Pinealectomized rats sensitized to ovalbumin demonstrated that pinealectomy significantly reduces the inflammatory cell counts in the bronchoalveolar lavage fluid after ovalbumin challenge, and that melatonin administration to pinealectomized rats restores the ability of inflammatory cells to migrate to the bronchoalveolar fluid. Those results suggest that melatonin may modulate the expression of chemotaxins in airway epithelial or endothelial cells[13].

The circadian variations of lung function in nocturnal asthma are associated with the increased airway inflammation during night. As a key regulator in human circadian rhythm homeostasis as well as an immunomodulator in allergic diseases, melatonin may regulate the circadian airway inflammation in asthma through modulating the expression of chemotaxins in the airway epithelial cells.

In order to test this hypothesis, we conducted the present study to answer two questions. First, whether melatonin is able to up-regulate RANTES and eotaxin expression in the lung epithelia cell line-A549. Second, what is the combinatory effect of melatonin and TNF-α on RANTES and eotaxin expression and whether this effect increases the eosinophils chemotactic activity (ECA) released in A549. The answers to these questions might provide new insights into the pathophysiology of asthma.

## Methods

This study was approved by the medical ethics committee of the West China Hospital of Sichuan University. Informed consents were obtained from all subjects in the study.

### Cell Culture

A549 cells, human type II-like epithelial lung cells, were obtained from ATCC (Manassas, VA, USA). The cells were cultured in tissue flasks incubated in 100% humidity and 5% CO_2 _at 37°C in DMEM medium (GIBCO BRL, Grand Island, NY) supplemented with 10% heat-inactived fetal bovine serum (GIBCO BRL) and penicillin-streptomycin (50 μg/ml, GIBCO BRL), at 1 × 10^6 ^cells/ml. A549 cells were then plated onto 6-well, flat-bottom tissue culture plates (Becton Dickinson and Co., NJ, USA) at a density of 1 × 10^6 ^cells/ well in DMEM medium. The medium was changed every 2 d until the cells became confluent and then the cells were used for the experiments.

### Cytokine Assays

As IL-1β and TNF-α have similar effect on the expression of many chemotaxins[14,15], we chose TNF-α as the representative pro-inflammatory cytokines in the asthmatic lung in this study. After the cells became confluent, the medium was changed to serum-free DMEM medium for 12 h. A549 cells were then exposed to increasing concentrations of melatonin (10^-10^, 10^-9^, 10^-8^, 10^-7^, 10^-6^M, the physiology concentration are 10^-9 ^to 10^-7 ^M during day and night[16]) (Sigma, St. Louis, MO, USA) and TNF-α (100 ng/ml) (Sigma), for 12 h. The cells were also stimulated with a combination of melatonin (10^-10^, 10^-9^, 10^-8^, 10^-7^, 10^-6^M) and TNF-α (100 ng/ml). The epithelial cell layers were then washed three times with Hanks' balanced salt solution (GIBCO BRL) and incubated for 48 h. Cell-free culture supernatants were collected. RANTES and eotaxin were assayed using enzyme-linked immunosorbent assay (ELISA) kits according to the instructions of the manufacturers. Assay kits for RANTES and eotaxin were purchased from R&D Systems (Minneapolis, MN, USA), and the minimum detectable concentration of RANTES and eotaxin was 5 pg/ml. Experiments were performed at least three times with the similar results.

### RNA extraction and real-time PCR

RNA extraction and real-time PCR were performed as previously described[17,18]. After the cells became confluent, the medium was changed to fetal bovine serum free DMEM medium for 12 h. A549 cells were then exposed to different concentrations of melatonin, together with or without TNF-α (100 ng/ml) (Sigma) for 12 h. Total cellular RNA was extracted using an acid guanidinium-phenol-chloroform method (Trizol; GIBCO BRL). RNA integrity was confirmed by electrophoresis on 1% agarose gels and ethidium bromide staining. Total cellular RNA, 1 μg, was reverse transcribed at 37°C for 70 min in 20 μl containing 2.5 U Superscript-II reverse transcriptase (GIBCO BRL); 10 mM dithiothreitol, 1 mM each of deoxyadenosine triphosphate (dATP), deoxythymidine triphosphate (dTTP), deoxycytidine triphosphate (dCTP), and deoxyguanidine triphosphate (dGTP); and 5 μg/ml oligo-dT primer (Pharmacia, Piscataway, NJ). Reactions were stopped by heat inactivation for 10 min at 85°C. Primers for human eotaxin, RANTES and β-actin were synthesized, HPLC purified as GIBCO BRL Custom Primers (Hong Kong, China). Primer sequences were as follows: Eotaxin: upstream primer: 5'- ACA TGA AGG TCT CCG CAG CAC TTC -3', downstream primer: 5'- TTG GCC AGG TTA AAG CAG CAG GTG -3'. RANTES upstream primer: 5'- GGC ACG CCT CGC TGT CAT CCT CA-3'; downstream primer: 5'- CTT GAT GTG GGC ACG GGG CAG TG-3'. β-actin upstream primer: 5'- AAG AGA GGC ATC CTC ACC CT -3',downstream primer 5'- TAC ATG GCT GGG GTG TTG AA -3'. Real-time PCR was performed on the ABI Prism 7700 sequence detection system (PE Applied Biosystems) by using SYBR green (Roche Diagnostics, Somerville, NJ) as a dsDNA-specific binding dye. The PCR were cycled 40 times after initial denaturation (95°C, 2 min) with the following parameters: denaturation, 95°C, 15s; and annealing and extension, 60°C, 1 min. The threshold cycle (CT) was recorded for each sample to reflect the mRNA expression levels. The fold changes of eotaxin or RANTES gene expression were calculated as previously described[18].

### Eosinophil Chemotaxis Assay

Eosinophil chemotaxis assay was performed as described previously[19]. Briefly, eosinophils were isolated from the peripheral blood of three healthy donors by negatively selected with immunomagnetic beads. Erythrocytes in venous peripheral blood were removed by hypotonic lysis. Neutrophils and mononuclear cells were depleted with anti-CD16 and anti-CD3 immunomagnetic beads (Miltenyi Biotec GmbH, Bergisch Gladbach, Germany). Eosinophils were stained with Randolph's stain and counted in a hemocytometer. Cytospins of each preparation were stained with Diff-Quik (International Reagent Corp., Green Cross, Osaka, Japan). The mean percentage of the eosinophil purity was 98.0 ± 0.3%. The viability measured by trypan blue exclusion was consistently greater than 95.0%. Eosinophil chemotaxis assay was measured by the Boyden's blind-well chamber technique using a 48-well, multiwell chamber (NeuroProbe Inc., Bethesda, MD). The bottom wells of the chamber were filled with 26.5 μl of the A549 cell supernatant stimulated by various chemicals, as described previously, in triplicate. A polycarbonate filter with a pore size of 5 μm (Nucleopore, Pleasanton, CA) was placed over the bottom wells, and isolated eosinophils were placed into each of the top wells. The chambers were then incubated at 37°C, 5% CO_2 _for 90 min. After incubation, eosinophils in the top wells were removed by scraping. The filter was then stained with Diff-Quik. Eosinophil chemotactic activity (ECA) is shown as the total number of migrated eosinophils counted in 10 high-power fields under a light microscope (Olympus, Lake Success, NY) at × 400 magnification.

### Data analysis

Data were expressed as means ± SD. Differences between groups were assessed by one-way ANOVA followed by the LDS significant difference test. A value of p < 0.05 was considered statistically significant.

## Results

### Effect of TNF-α and melatonlin on RANTES and eotaxin released from A549 cells

**Figure 1:**
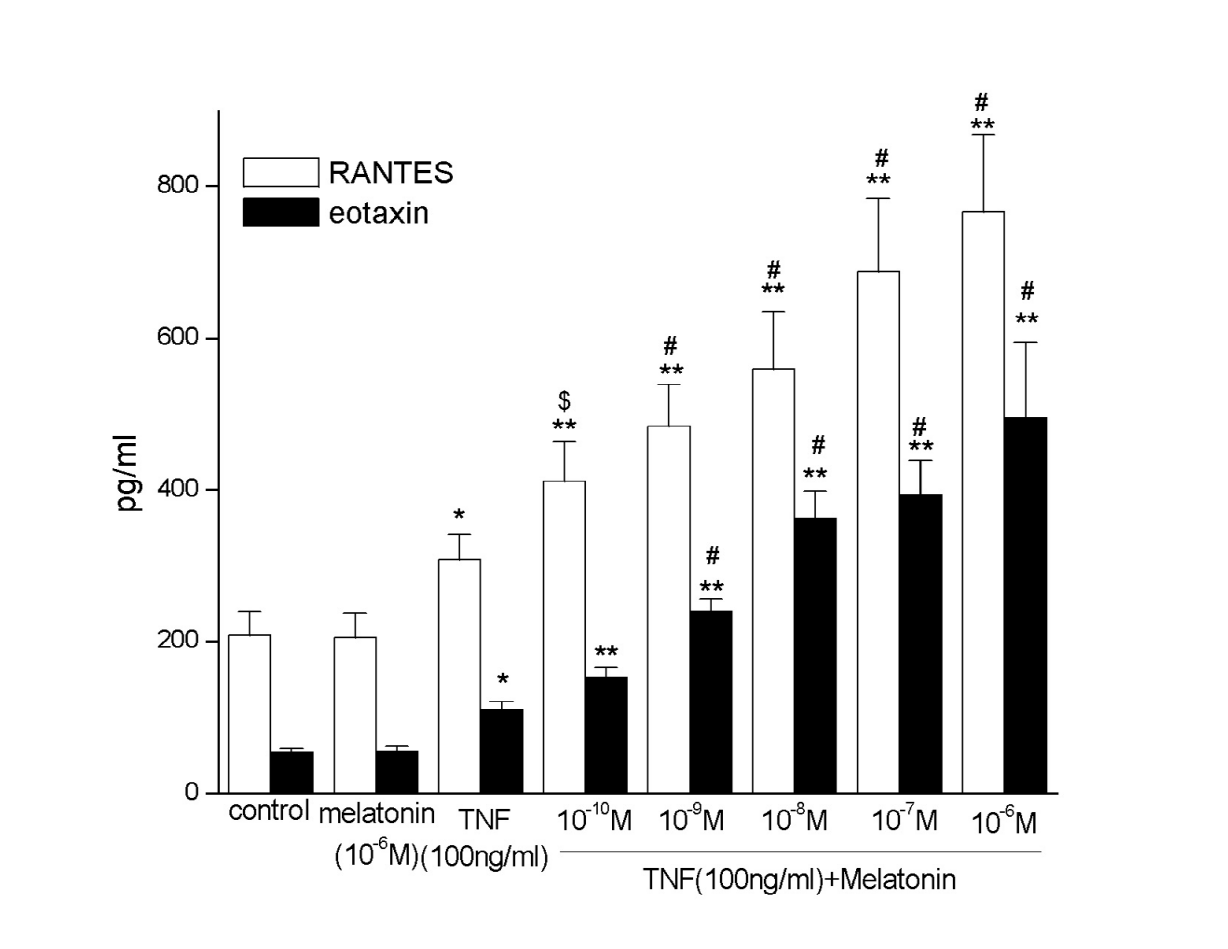
**RANTES and eotaxin released from A549 cells. **Melatonin(10^-6^M) alone did not change RANTES and eotaxin released from A549 cells. However, it (from10^-10 ^to 10^-6^M) promoted RANTES and eotaxin released from A549 cells in a dose dependent manner when co-stimulated with TNF-α (100 ng/ml). * and **, p < 0.05 and 0.01, compared with control and melatonin alone (pg/ml, n = 3). $ and #, p < 0.05 and 0.01, compared with TNF-α alone (pg/ml, n = 3).

RANTES released from A549 cells increased significantly when the cells incubated with TNF-α(100 ng/ml). Melatonin alone didn't have this effect on A549 in dose from10^-10 ^to 10^-6^M. However, TNF-α induced RANTES release in A549 increased significantly by incubation with melatonin (from10^-10 ^to 10^-6^M). Similarly, eotaxin released from A549 cells also increased significantly when the cells incubated with TNF-α; Melatonin alone had no effect on eotaxin released from A549 at dose range from10^-10 ^to 10^-6^M. However, eotaxin released from A549 increased significantly when the cells incubated with melatonin and TNF-α (Figure 1).

### Effect of TNF-α and melatonlin on the expression of RANTES and eotaxin in A549 cells

**Figure 2:**
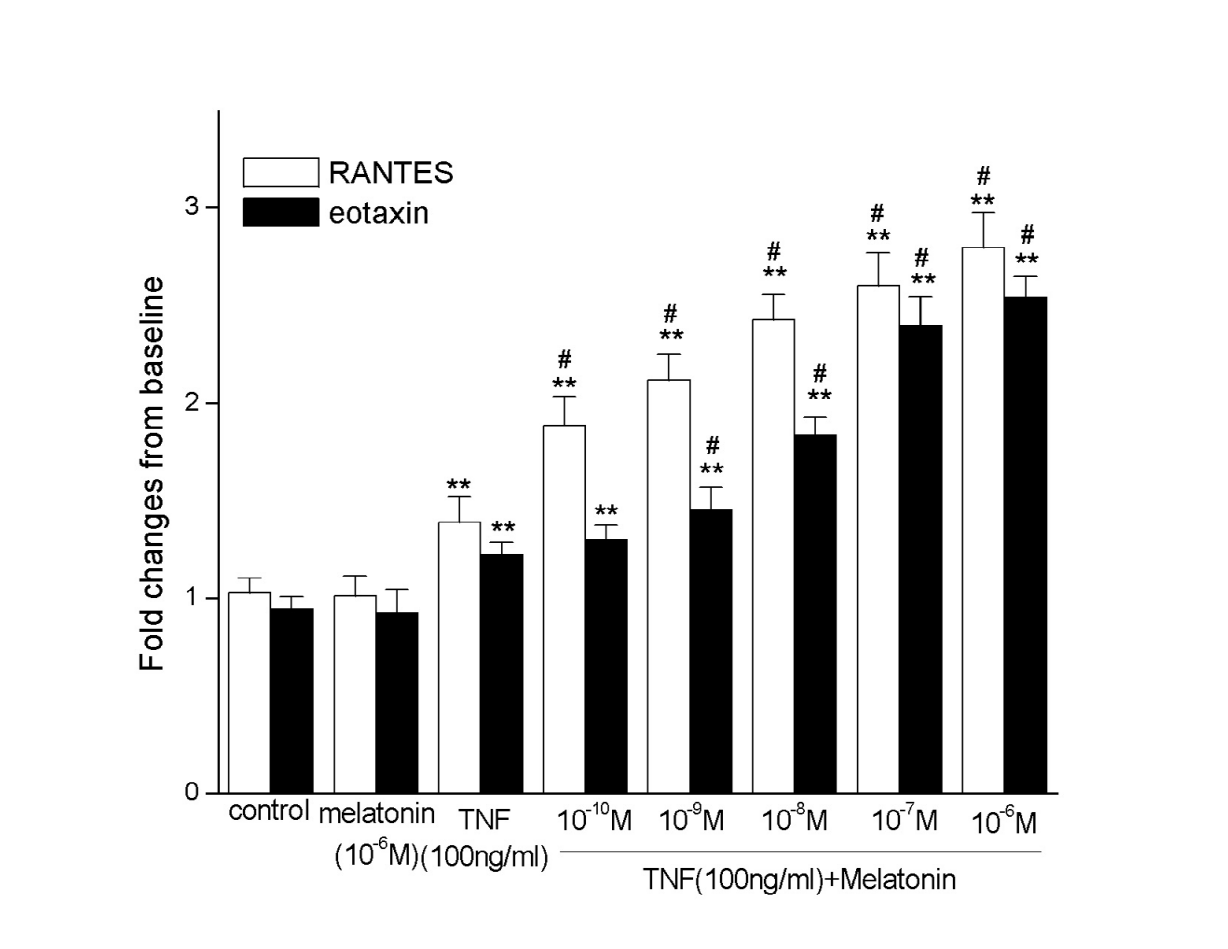
**RANTES and eotaxin mRNA expression in A549 cells. **Melatonin(10^-6^M) alone did not change the RANTES and eotaxin mRNA expression in A549 cells. TNF-α (100 ng/ml) could promote the RANTES and eotaxin expression in A549 cells. Melatonin (from10^-10 ^to 10^-6^M) increased the RANTES expression of A549 cell in a dose dependent manner when co-stimulated with TNF-α(100 ng/ml). **, p < 0.01, compared with control and melatonin alone (n = 3). #, p < 0.01, compared with TNF-α alone (n = 3).

To determine whether the production of RANTES and eotaxin is accompanied by the transcription of the corresponding genes, we used real-time RT-PCR to examine RANTES and eotaxin mRNA expression in A549 cells. A549 were stimulated with melatonin (10^-10^, 10^-9^, 10^-8^, 10^-7^, 10^-6^M) and TNF-α (100 ng/ml). Melatonin alone did not change the RANTES and eotaxin mRNA expression in A549. TNF-α can promote the RANTES and eotaxin expression in A549 cells. When stimulated with TNF-α, melatonin synergistically increased the RANTES and eotaxin expression in a dose dependent manner (Fig 2).

### Effect of TNF-α and melatonlin on eosinophil chemotactic activity (ECA) released by A549 Cells

**Figure 3:**
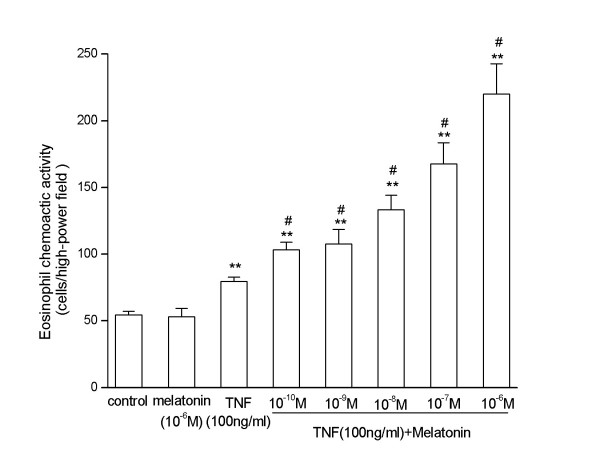
**Eosinophil chemotactic activity (ECA) released from A549 cells. **Melatonin (10^-6^M) alone did not change the ECA released from A549 cells. TNF-α (100 ng/ml) could increase the ECA released from A549 cells. Melatonin (from10^-10 ^to 10^-6^M) increased the ECA released from A549 cell in a dose dependent manner when co-stimulated with TNF-α(100 ng/ml). **, p < 0.01, compared with control and melatonin alone (n = 3). #, p < 0.01, compared with TNF-α alone (n = 3).

When stimulated with TNF-α(100 ng/ml), ECA released by A549 cells increased significantly. Melatonin (from10^-10 ^to 10^-6^M) alone didn't have this effect. When stimulated with TNF-α (100 ng/ml) and melatonin, ECA released increased in A549 cells in a dose dependent manner (Fig 3).

## Discussion

In this study, we examined the RANTES and eotaxin protein level and the gene expression in A549 in response to TNF-α and melatonin stimulation using ELISA and real-time RT-PCR. We also measured the ECA released by A549 in response to TNF-α and melatonin stimulation. Unexpected, we found that the eotaxin and RANTES protein level and gene expression in A549 cells were unchanged when treated with melatonin alone, and the ECA released by A549 remained unchanged too. However, when A549 cells co-stimulated with melatonin and TNF-α, eotaxin and RANTES released from the cells increased in a melatonin dose dependent manner. The gene expression of eotaxin and RANTES, and the ECA also increased at the same time. This result support our hypothesis that melatonin play an important role in airway inflammation through up-regulation of the eotaxin and RANTES expression in lung epithelial cell when the cells stimulated with pro-inflammatory cytokines.

The pro-inflammatory characteristics of TNF-α have been documented extensively. Numerous studies have demonstrated that these attributes contribute to the inflammatory conditions present in airways of asthmatic subjects. TNF-α has been shown to activate the inflammatory cells, up-regulate the adhesion molecules on endothelium and circulating leukocytes, increase the production of chemotaxins[20], the bronchial responsiveness. TNF-α is expressed primarily by the alveolar cells and tissue macrophages, mast cells, and bronchial epithelial cells. Additionally, in most other airway cell systems studied, conditions simulating an inflammatory state result in expression of TNF-α. Thus, it is not surprising that TNF-α concentration is higher in the bronchoalveolar lavage fluid from symptomatic asthmatics compared with normal control subjects[21]. In this study, we found that TNF-α could promote the RANTES and eotaxin production in A549 and melatonin further exaggerated this effect of TNF-α.

Lung function in a healthy individual varies in a circadian rhythm, with the peak lung function occurring near 4:00 PM (1600 hours) and the minimal lung function occurring near 4:00 AM (0400 hours). An episode of nocturnal asthma is characterized by an exaggeration in this normal variation in lung function from daytime to nighttime, with diurnal changes in the pulmonary function generally of > 15%. A recent study showed that the circadian variability in pulmonary function in asthma was related to changes in the airway eosinophils recruitment and activation[22]. Although the molecular mechanism responsible for the selective infiltration of eosinophils into the inflamed tissue in asthma has not been elucidated, chemotaxin may play an important role in this process. Eotaxin is a chemotaxin that binds with high affinity and specificity to the chemotaxin receptor CCR3 and plays an important role in the pathogenesis of allergic disease. RANTES, a C-C chemotaxin, was initially shown to be chemoattractant for T cells and monocytes but has subsequently been shown to be a potent eosinophil chemoattractant[23,24]. In other studies, an up-regulation of RANTES message was observed in the airways of asthmatic patients[25], and increased levels of RANTES have been detected in the nasal aspirates of children with the viral exacerbation of asthma[26], suggesting an important role for RANTES in this process. From the result of our study, together with the studies above, we can infer that melatonin, the most important circadian rhythm regulator, may also regulate the asthma airway inflammation by up-regulating the expression of eotaxin and RANTES in the airway epithelium in inflammatory status of asthma.

RANTES and eotaxin expression are regulated by two important transcriptional factors: active protein-1 (AP-1) and nuclear factor kappa B(NFκB). Benis et al[27] found that melatonin could suppress the activation of NFκB and AP-1. Although NFκB and AP-1 could up-regulate the expression of many pro-inflammatory cytokines and chemotaxins, other transcriptional factors also could be involved in the regulation of RANTES and eotaxin. Further studies are needed to elucidate the mechanism of how melatonin regulates the transcription of these chemotaxins.

The role of melatonin as an immunomodulator is poorly understood and, in some cases, contradictory results have been reported. For example, Shafer's study showed that melatonin has no effect on the activity of stimulated macrophages[28]. However, pinealectomy of rats significantly reduces airway inflammation after ovalbumin inhalational challenge, and melatonin administration to the pinealectomized rats seems to restore the airway inflammation, which further supports the pro-inflammatory effect of melatonin. In addition, up-regulation of the gene expression of transforming growth factor-β(TGF-β), macrophage-colony stimulating factor (M-CSF), TNF-α and stem cell factor (SCF) in peritoneal exudate cells, and up-regulation of the gene expression of IL-1β, M-CSF, TNF-α, interferon-γ (IFN-γ) and SCF in splenocytes, were observed in male C57 mice received 10 consecutive daily intraperitoneal injections of melatonin[12]. Further research should be directed at evaluating the mechanism of melatonin regulating the transcription of those kinds of cytokines.

## Conclusion

Melatonin alone did not change eotaxin and RANTES protein level and gene expression in A549 cells, and had no effect on ECA released by A549 cells. However, when A549 cells were stimulated with melatonin, together with TNF-α, the mRNA expression and protein release of eotaxin and RANTES increased significantly. This result suggested that combined with pro-inflammatory cytokines, melatonin may play a role in the airway inflammation through up-regulation of the eotaxin and RANTES expression in the lung epithelial cells.

## Authors' contributions

FML conceived of the experiment, carried out all experiments and prepared the manuscript. XJL conceived of the experiment and performed RNA extraction and real-time RT-PCR. SQL conceived of the experiment and assisted in collection and analysis of ELISA samples. CTL performed cell culture and provided expert advice and interpretation of the study's results. WZL participated in the study's design, coordination and final revisions of the manuscript. All authors read and approved the final manuscript.
